# Application of 4-CPA or ethanol enhances plant growth and fruit quality of *phyA* mutant under heat stress

**DOI:** 10.1038/s41598-025-17929-8

**Published:** 2025-09-12

**Authors:** Riham A. H. Ahmed, Islam M. Y. Abdellatif, Natsumi Oka, Misaki Kobayashi, Martina Bianca Fuhrmann-Aoyagi, Daisuke Todaka, Motoaki Seki, Kenji Miura

**Affiliations:** 1 Degree Programs in Life and Earth Sciences, University of Tsukuba, Tsukuba, 305-8572 Japan; 2 Tsukuba-Plant Innovation Research Center, University of Tsukuba, Tsukuba, 305-8572 Japan; 3 Department of Horticulture, Faculty of Agriculture, Minia University, El-Minia, 61517 Egypt; 4 Plant Genomic Network Research Team, RIKEN Center for Sustainable Resource Science, Yokohama, Kanagawa Japan; 5 Kihara Institute for Biological Research, Yokohama City University, Yokohama, Kanagawa Japan

**Keywords:** Plant breeding, Plant stress responses

## Abstract

As the phytochromes play a key role in plant light perception, they also modulate stress responses. The tomato mutant c.v. Moneymaker lacking *PHYTOCHROME A* (*PHYA*) exhibits tolerance to heat stress during the vegetative growth phase; however, this response does not continue into the reproductive stage. In this study, the response of *phyA* at the reproductive stage was improved through the exogenous application of 4-chlorophenoxy acetic acid (4-CPA) and ethanol under heat stress, either at 37 °C in controlled culture room or fluctuating high temperatures (approximately 50 °C and 30 °C in midday and night, respectively) during the summer in greenhouse. 4-CPA, a synthesized plant growth regulator with functions similar to that of auxins, induced growth and flower formation at the flowering stage when sprayed on *phyA* compared to that in the non-treated plants. Similarly, 4-CPA application improved fruit setting and fruit characteristics, and the quantity or quality of the *phyA* mutant. The expression of numerous heat-related genes, such as heat shock factors (*HSFs*), heat shock proteins (*HSPs*), and reactive oxygen species (ROS) scavengers, was upregulated in *phyA* as a result of 4-CPA application. Ethanol application showed better growth when sprayed on *phyA* than non-treated plants, and *HSFA1a* and *HSP70* expression was significantly stimulated by this treatment. On the other hand, applying either 4-CPA or ethanol-induced auxin and gibberellin production by stimulating the expression of genes involved in hormone production. Finally, electrolyte leakage (EL) and malondialdehyde (MDA) levels were inhibited after both treatments. In contrast, proline production increased. Therefore, applying 4-CPA or ethanol improved *phyA* mutant tolerance, allowing the mutant to tolerate heat stress during all growth stages. Furthermore, the quality of tomato fruits is improved by the application of these chemicals.

**Supplementary Information:**

The online version contains supplementary material available at 10.1038/s41598-025-17929-8.

## Introduction

Among plant photoreceptors, PHYTOCHROMEs (PHYs) are the well-characterized and are essential for regulating key developmental processes, including germination, de-etiolation, and flowering^1,2^. The number and types of *PHYs* vary across plant species. For instance, both the tomato and Arabidopsis genomes contain five *PHY* genes; however the types differ with *PHYA*, *B1*, *B2*, *E*, and *F* found in tomatoes^3^ and *PHYA*, *B*, *C*, *D*, and *E* in Arabidopsis^4^. Rice contains only three *PHYs* (*PHYA*, *B*, and *C*)^5^.

The tomato *phytochrome A* (*phyA*) mutant exhibited thermotolerance, particularly during the vegetative growth stage, characterized by enhanced membrane stability, reduced electrolyte leakage (EL) and malondialdehyde (MDA) accumulation, and increased proline levels, contributing to the osmotic balance and stress adaptation. These physiological traits were supported by the upregulation of stress-responsive genes, such as *HSFA2*, *HSFB1*, and *GRP*, which are associated with improved heat resistance. However, the *phyA* mutant displays defects during the fruiting stage and produces smaller parthenocarpic fruits. Unlike the WT, the *phyA* mutant failed to stimulate the expression of heat shock factors (*HSFs*) and heat shock proteins (*HSPs*), which are important for stress adaptation^6^. Improving the growth and productivity of the *phyA* mutant during the reproductive stage is a promising area for future research.

4-Chlorophenoxy acetic acid (4-CPA) is an auxin that influences fruit setting in tomatoes. In particular, applying 4-CPA after anthesis increased the percentage of fruit set, number of fruits per plant, weight, fruit diameter, number of fruits per cluster, and yield per cluster at lower temperatures^7^. 4-CPA is a plant growth regulator that enhances fruit set during summer^8^, and can also induce parthenocarpy and fruit development without fertilization^9^. When the synthetic auxin 4-CPA was applied to flowers, it successfully induced parthenocarpic ovarian growth^9^.

Chemical priming is a useful strategy for improving plant stress resistance^10^. Various chemical compounds can activate the molecular processes that regulate tolerance to environmental stress. Recent studies have demonstrated that applying ethanol to plants can improve their tolerance to environmental stresses. Ethanol is widely available and is considered environmentally- and human-friendly^11^. It has emerged as a promising agent for enhancing plant stress tolerance as an alternative to transgenic approaches and traditional breeding methods. It mitigates drought stress in Arabidopsis and improves drought tolerance in wheat and rice^12^. Application of 20 mM ethanol improved growth in drought-stressed soybean plants by increasing biomass, leaf area per trifoliate, gas exchange features, water-use efficiency, photosynthetic pigment content, and leaf relative water content^13^. Additionally, ethanol enhances tolerance to high-salinity stress by detoxifying reactive oxygen species (ROS) in both Arabidopsis and rice^14^. It also increases heat stress tolerance in Arabidopsis by activating unfolded protein response signaling, which is facilitated by putrescine accumulation^15^. Ethanol treatment alleviates salt stress in soybean plants by boosting ROS detoxification mechanisms, enhancing antioxidant enzyme activity, and promoting osmotic adjustment by increasing proline and amino acid levels^16^. Moreover, ethanol reduces oxidative damage caused by stress in *Arabidopsis * by suppressing ROS accumulation^17^. Ethanol-induced heat stress tolerance in tomatoes is mainly the result of increased expression of stress-related genes encoding late embryogenesis-abundant (LEA) proteins, ROS elimination enzymes, and activated gluconeogenesis^11^. Ethanol stimulates the unfolded protein response, making plants more heat tolerant^15^.

The aim of this study was to identify the *phyA* response to heat stress during the reproductive stage after exogenous application of 4-CPA and ethanol. Because the *phyA* mutant exhibited heat tolerance in vegetative growth stage^6^, if application of 4-CPA and ethanol enhance heat tolerance in reproductive growth stage, combination of the *phyA* mutation and chemical treatments enhances heat tolerance in both stages. 4-CPA and ethanol improved plant growth of the *phyA* mutant under heat stress, either at 37 °C or fluctuating high temperatures during the summer in greenhouse conditions. Furthermore, both chemical treatments improved fruit quality, such as increase in sugar content and ascorbic acid. These results indicate that tolerance to heat stress in both vegetative and reproductive growth stages is improved by combination of the *phyA* mutation and chemical treatments.

## Results

### Enhancement of heat tolerance by applying 4-CPA and ethanol

**Fig. 1:**
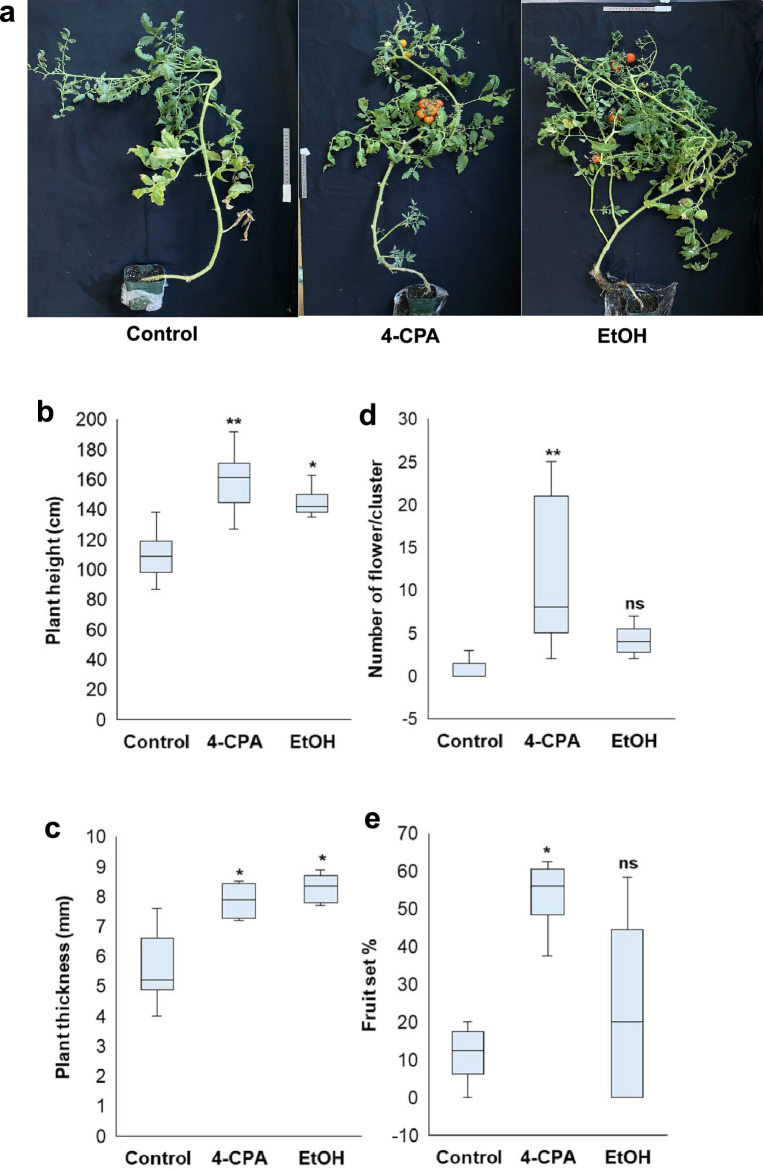
Morphological response under heat stress treated with 4-CPA or ethanol (EtOH). (**a**) Plant phenotype under 37 °C. Several parameters, such as plant height (**b**), plant thickness (**c**), number of flower/cluster (**d**), and rate of fruit set (**e**) were measured. Statistical analysis was performed to determine significant differences compared to the control at *p* < 0.05 (*) and at *p* < 0.01 (**) (n ≥ 4).

Under HS conditions at 37°C in controlled cultivation room and in greenhouse environments (approximately 50 °C and 30 °C in midday and night, respectively), applying 4-CPA and ethanol significantly affected plant growth. Plants treated with 4-CPA and ethanol exhibited improved phenotypic traits under stress conditions, including enhanced height and stem thickness, compared to that of the control plants (Fig. 1a–c, Supplementary Fig. S1a). These results indicated that 4-CPA and ethanol application improved plant tolerance to HS.

Tomato flowers are highly susceptible to HS, which can cause morphological changes in flower structure^18^. Observations under stable high-temperature conditions (37 °C) revealed that flowers treated with 4-CPA developed faster than those in the control and ethanol-treated groups (Supplementary Fig. S1a).

**Fig. 2:**
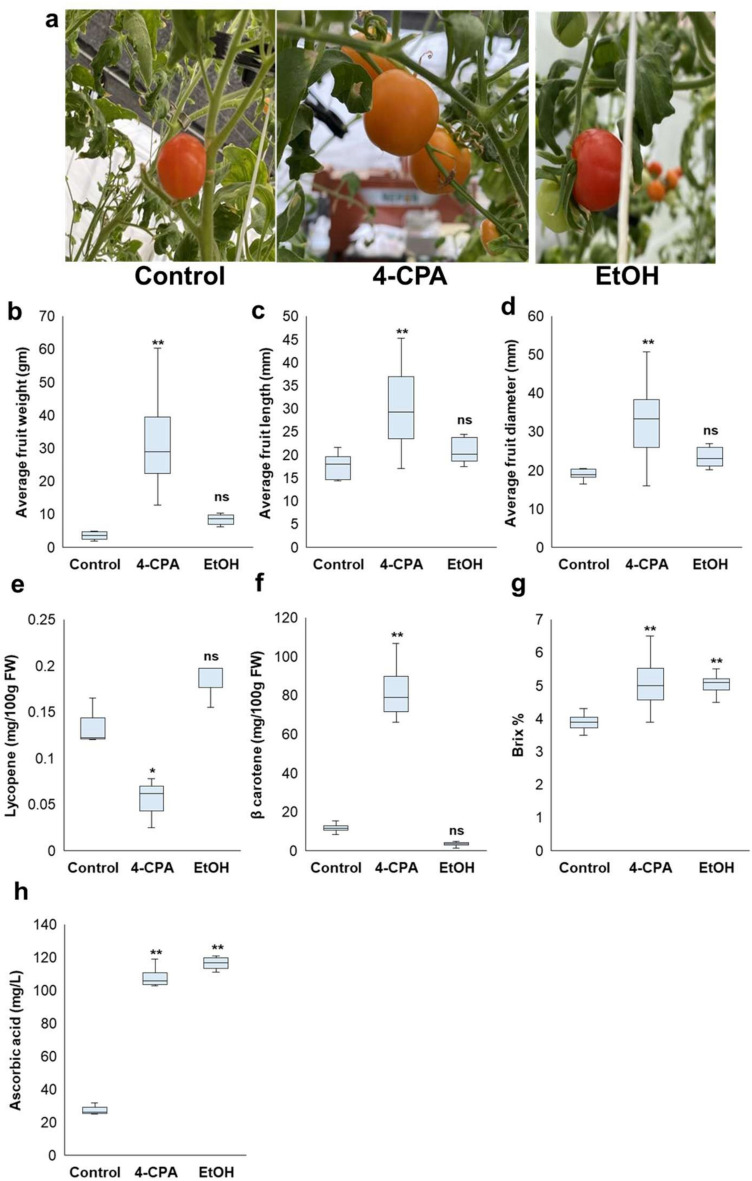
Fruit quality under heat stress. (**a**) Fruit phenotype and color. Application of 4-CPA exhibited orange-colored fruits, but others did red-colored fruits. Morphological parameters, such as fruit weight (**b**), fruit length (**c**), and fruit diameter (**d**) were investigated. The contents of lycopene (**e**) or *β*-carotene (**f**) in 4-CPA-treated tomato fruits was decreased or increased, respectively, leading to the orange-colored fruits. Brix (**g**) and ascorbic acid (**h**) levels were increased in both 4-CPA and EtOH. Statistical analysis was performed to determine significant differences compared to the control at *p* < 0.05 (*) and at *p* < 0.01 (**) (n ≥ 3).

Applying 4-CPA has been reported to increase fruit set, yield, and economic benefits during summer tomato production. In the present study, 4-CPA application significantly increased the number of flowers / clusters, and these flowers successfully developed into fruits under stressful conditions (Fig. 1d). This indicated that 4-CPA application improved fruit set (Fig. 1e). Although ethanol application also enhanced flower development and fruit set, the differences observed were not statistically significant compared with the control (Fig. 1e). Fruit characteristics, including weight, length, and diameter, were measured under HS conditions. Applying 4-CPA increased fruit weight, length, and diameter significantly compared to those of the control. Ethanol application also improved these fruit characteristics; however, the differences were not statistically significant compared to the control treatment (Fig. 2a–d).

### Fruit quality after 4-CPA and ethanol application

Fruit quality was evaluated by measuring key parameters, including total soluble solids (Brix %), ascorbic acid content, lycopene, and *β*-carotene levels, for both applications. Brix increased significantly with the 4-CPA and ethanol treatments (Fig. 2g). To confirm increase in Brix by ethanol application, another cultivar (Sicilian Rouge) was used. Application of ethanol also increased Brix value although other characteristics, such as fruit weight, diameter, and length, were unchanged (Supplementary Fig. S2).

Additionally, the ascorbic acid content increased with both applications (Fig. 2h). Differences in the tomato color were observed after 4-CPA application compared to other treatments, prompting an analysis of lycopene and *β*-carotene content in the fruits. The results showed that application with 4-CPA inhibited lycopene accumulation while significantly increasing *β*-carotene levels compared to the control and ethanol-treated fruits. This explained the orange coloration observed in the tomato fruits treated with 4-CPA.

### Physiological responses to chemical treatments under HS

The physiological responses of the plant were evaluated under three conditions, i.e., normal conditions (25 °C), 37 °C, and greenhouse conditions. The plants were subjected to these conditions for 3 weeks for the HS treatment.

**Fig. 3:**
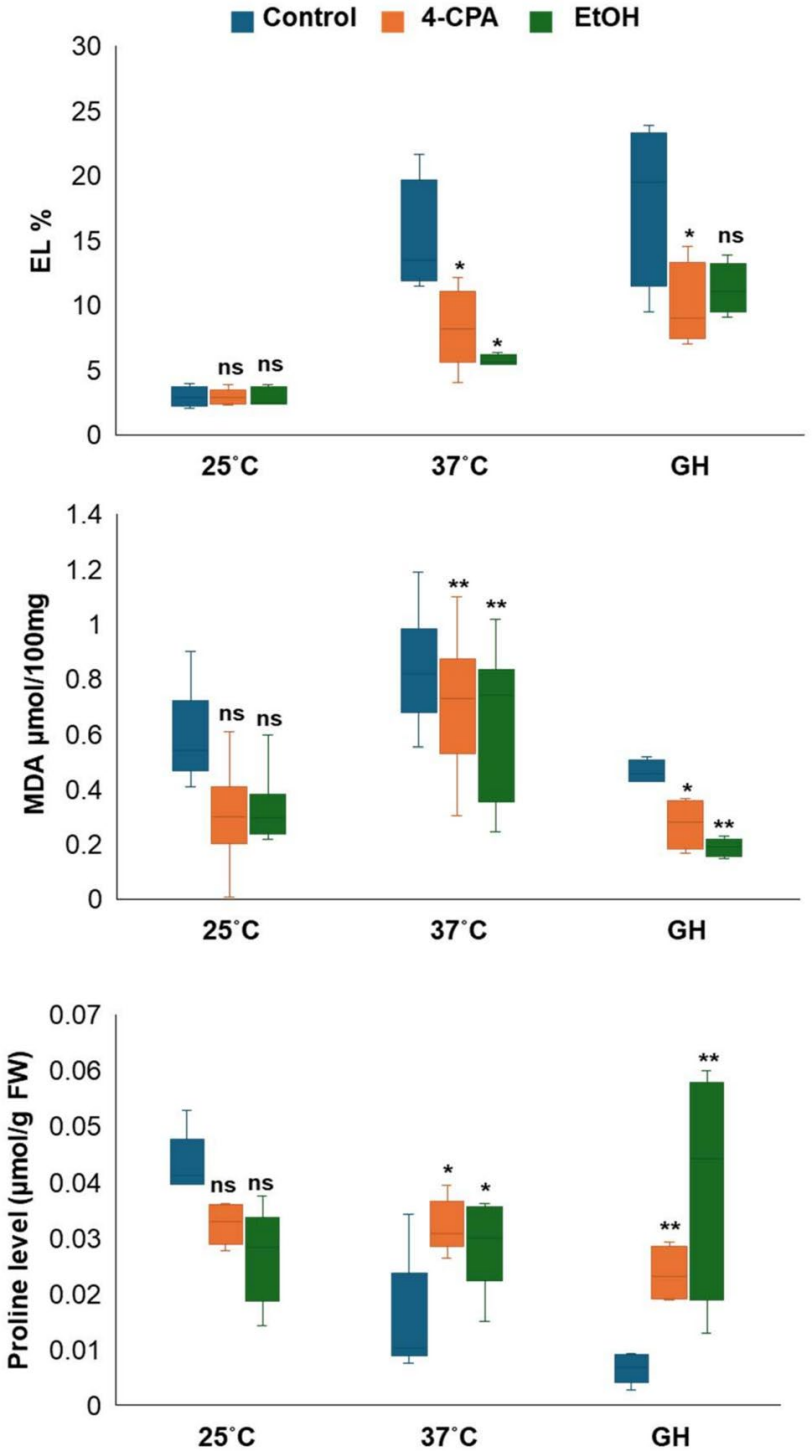
Physiological analysis under three conditions, at 25 °C, at 37 °C, and under greenhouse conditions. Electrolyte leakage (EL) (**a**), malondialdehyde (MDA) accumulation (**b**), and proline level (**c**) were investigated. Statistical analysis was performed to determine significant differences compared to the control at *p* < 0.05 (*) and at *p* < 0.01 (**) (n ≥ 4).

Membrane thermostability is a reliable parameter for assessing plant tolerance to HS. The membrane stability of the *phyA* mutant following 4-CPA and ethanol treatment was investigated by measuring the EL of tomato leaves. At 37 °C and under greenhouse environments, plants treated with 4-CPA and ethanol exhibited the lowest EL values, which were significantly lower than those of the control. However, there was no significant difference in the EL between the chemical treatments and the control under normal conditions (Fig. 3a).

MDA is an end product of polyunsaturated fatty acid peroxidation in cells and serves as a marker of oxidative stress. There were no significant differences in the MDA levels among the control, 4-CPA, and ethanol treatments under normal conditions. However, both applications significantly reduced MDA accumulation at 37°C and under greenhouse conditions compared to the control (Fig. 3b). These findings indicated that 4-CPA and ethanol application enhanced the membrane thermostability of green organs and inhibited membrane lipid peroxidation under HS during the reproductive stage in the *phyA* mutant.

Proline is a key abiotic stress indicator in plants and contributes to stabilizing the subcellular structure and scavenging of free radicals. There were no significant differences in proline levels between the control and the two treatments under normal conditions. However, the 4-CPA and ethanol applications resulted in the highest proline accumulation at 37°C and under greenhouse conditions (Fig. 3c).

### Changes in stomata features under HS conditions with 4-CPA and ethanol applications

**Fig. 4:**
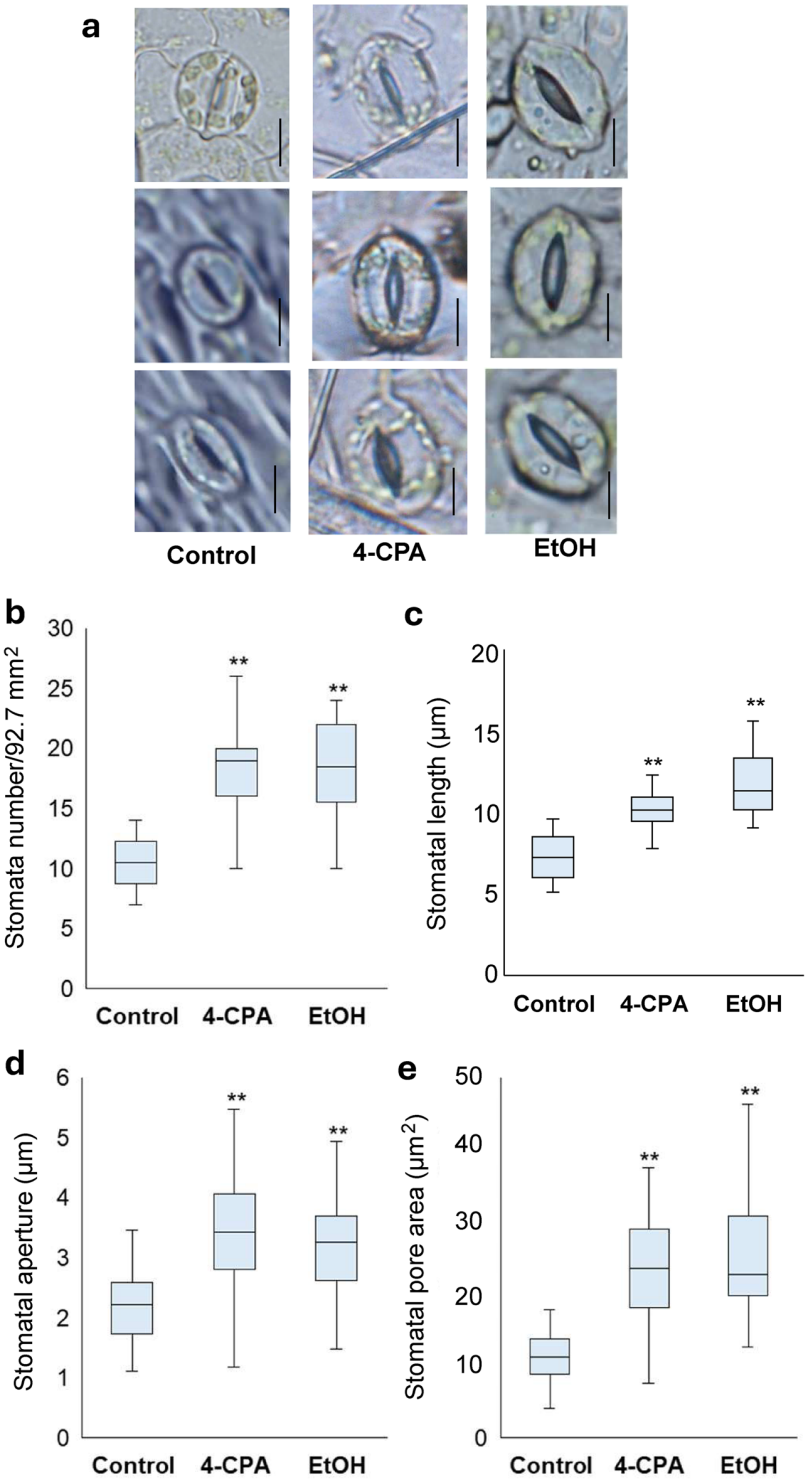
Stomata response under heat stress. (**a**) Stomata under heat stress treated with 4-CPA or EtOH were observed with microscope. Bars indicate 5 μm length. Stomatal phenotypes, such as stomata number (**b**), stomatal length (**c**), stomatal aperture (**d**), and stomatal pore area were investigated. Statistical analysis was performed to determine significant differences compared to the control at *p* < 0.01 (**) (n ≥ 16).

Stomata are small pores in the aboveground organs of plants that facilitate gas exchange and water regulation between plants and their surrounding environment. Their development is highly sensitive to environmental fluctuations, including temperature stress^19^. Stomata number, stomatal pore length, diameter, and area were measured in plants grown at 37°C. The stomatal number of 4-CPA- and ethanol-treated plants was significantly higher than that of the control plants (Fig. 4b, Supplementary Fig. S3). Additionally, stomatal pore length and diameter were significantly larger in the treated plants, leading to an increase in stomatal pore area relative to that of the control (Fig. 4).

Fertile pollen directly contributes to crop productivity and is essential for the survival, fitness, and dispersal of flowering plants^20^. Pollen fertility was assessed in flowers from 4-CPA and ethanol-treated plants compared to the control plants, 2 weeks after exposure to HS at 37°C. There were no significant differences in pollen viability among the treatments, and high viability was observed in all groups (Supplementary Fig. S4a), suggesting that pollen viability was not significantly affected by HS.

The pollen tube growth was investigated in vivo. Temperatures above 30°C impair tomato pollen germination and pollen tube growth^21^. Pollen tube germination was observed 24 h after hand-pollination of the stigma at 25 °C. Although all pollen showed high viability under stress, the pollen tubes from the control and ethanol-treated plants did not grow, whereas only pollen from the 4-CPA-treated plants grew under HS (Supplementary Fig. S4b), indicating that 4-CPA application promoted pollen tube growth under HS.

### 4-CPA and ethanol treatment enhanced expression of *HSFs, HSPs,* and several hormone-related genes under HS during the fruiting stage

**Fig. 5:**
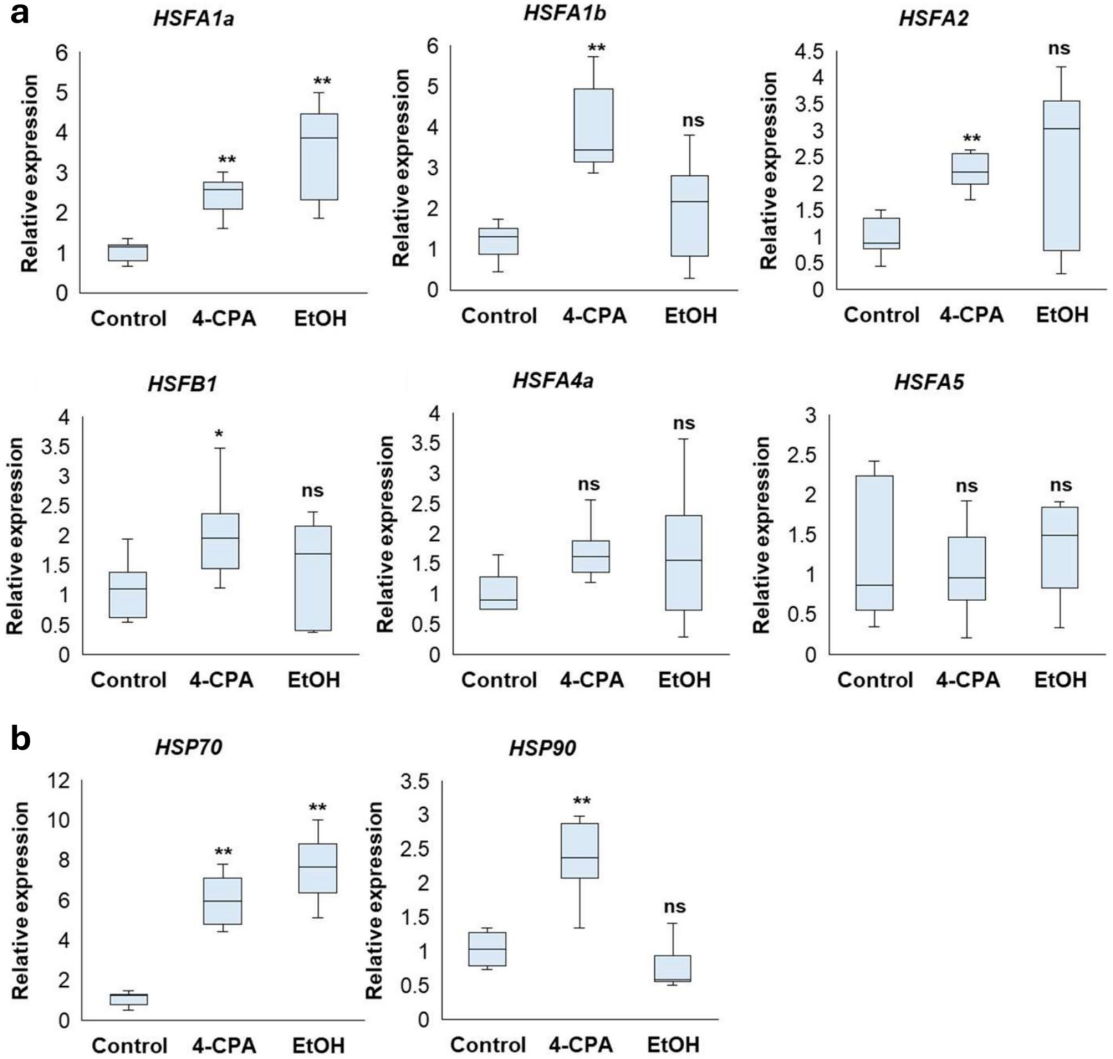
Expression level of heat shock transcription factors (*HSFs*) genes and heat shock protein (*HSPs*) genes under heat stress treated with 4-CPA or EtOH. The expression levels of *HSFs* (*HSFA1a*, *HSFA1b*, *HSFA2*, *HSFB1*, *HSFA4a*, and *HSFA5*) (**a**) and of *HSPs* (*HPS70* and *HSP90*) (**b**) were examined. Statistical analysis was performed to determine significant differences compared to the control at *p* < 0.01 (**) (n ≥ 3).

HS reduces the efficiency of plant physiological and biochemical processes by influencing molecular mechanisms. The ability of plants to tolerate various abiotic stresses, including HS, is considerably influenced by HSFs and HSPs^22^. The expression levels of heat-responsive genes, including *HSFs* and *HSPs*, were examined in control plants and in plants treated with 4-CPA or ethanol under greenhouse conditions. The expression levels of several *HSF* genes, such as *HSFA1a*, were upregulated in both 4-CPA- and ethanol-treated plants compared to those in the control, while *HSFA1b, HSFA2,* and *HSFB1* were upregulated after 4-CPA treatment. The expression levels of *HSFA4a* and *HSFA5* did not differ significantly across the treatments (Fig. 5a). Additionally, the expression of *HSP70* was upregulated in both 4-CPA- and ethanol-treated plants compared to in the control, whereas the expression of *HSP90* significantly increased only in response to 4-CPA treatment (Fig. 5b). These results suggested the involvement of HSFs and HSPs in heat response mechanisms activated by 4-CPA or ethanol.

**Fig. 6:**
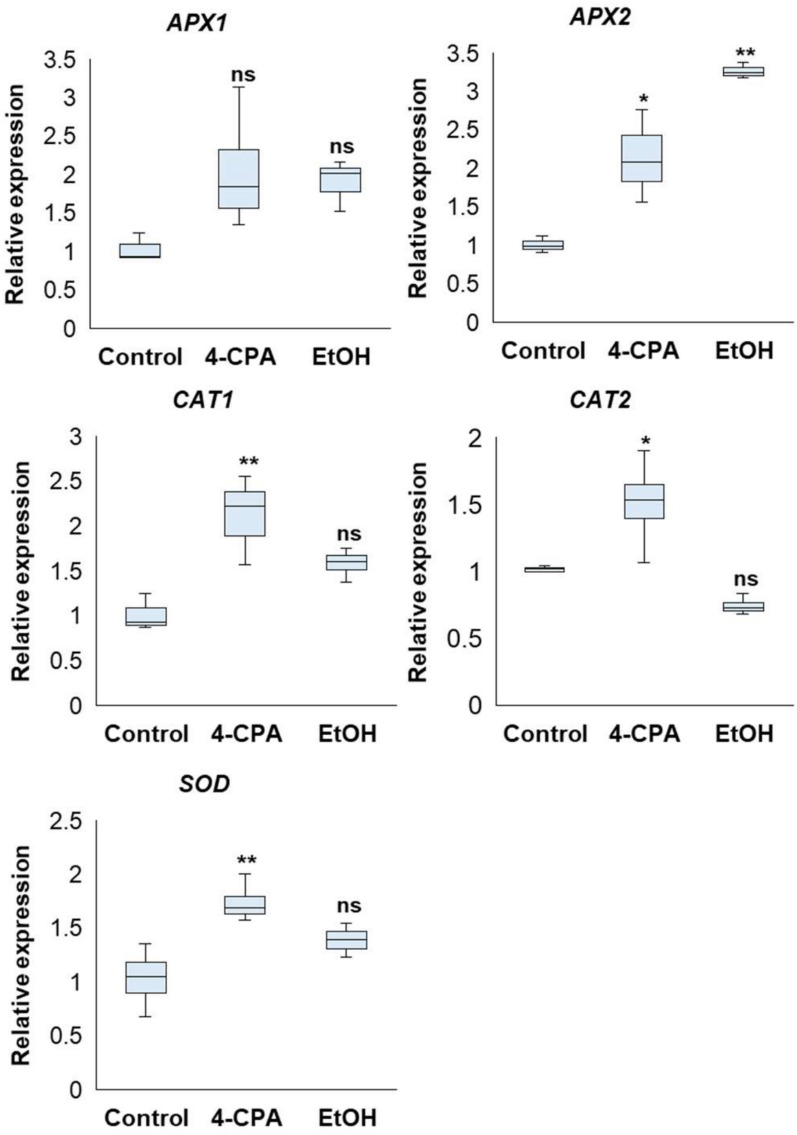
Expression level of enzymes for antioxidant defense systems, such as ascorbate peroxidase (*APX*), catalase (*CAT*), and superoxide dismutase (*SOD*) under heat stress treated with 4-CPA or EtOH. Statistical analysis was performed to determine significant differences compared to the control at *p* < 0.05 (*) and at *p* < 0.01 (**) (n ≥ 3).

Ascorbate peroxidase (*APX*), catalase (*CAT*), and superoxide dismutase (*SOD*) are enzymatic antioxidant defense systems in plants against environmental stress factors that are essential for scavenging ROS and reducing oxidative stress^23^. In this study, the expression levels of *APX1*, *APX2*, *CAT1*, *CAT2*, and *SOD* were measured after exposure to HS. The expression of *APX1* did not show a significant increase in 4-CPA- or ethanol-treated *phyA* plants compared to non-treated plants (Fig. 6). *APX2* expression was significantly stimulated by both chemical treatments compared to that in the non-treated plants (Fig. 6). A similar result was observed for the expression of *CAT1* and *CAT2*, in which 4-CPA-treated *phyA* showed significant upregulation compared to the ethanol or non-treated group (Fig. 6). *SOD* expression was similar to the *CAT* genes expression results, and 4-CPA application significantly stimulated *SOD* expression compared to the other treatments (Fig. 6). These results indicated that *phyA* plants treated with 4-CPA stimulated expression of *APX2*, *CAT1*, *CAT2*, and *SOD* for ROS scavenging, whereas ethanol stimulated *APX2*.

Tomato contains a single *euAP3* lineage gene, *Tomato APETALA3* (*TAP3*), and *Tomato MADS-box* gene 6 lineage (*TM6*), both of which play distinct roles in floral development. *TAP3* is essential for specifying petal and stamen identities, whereas *TM6* is crucial for stamen differentiation^24^. The expression level of *TM6* significantly increased in both treatments (Supplementary Fig. S5), indicating that both treatments enhanced floral organ development.

**Fig. 7:**
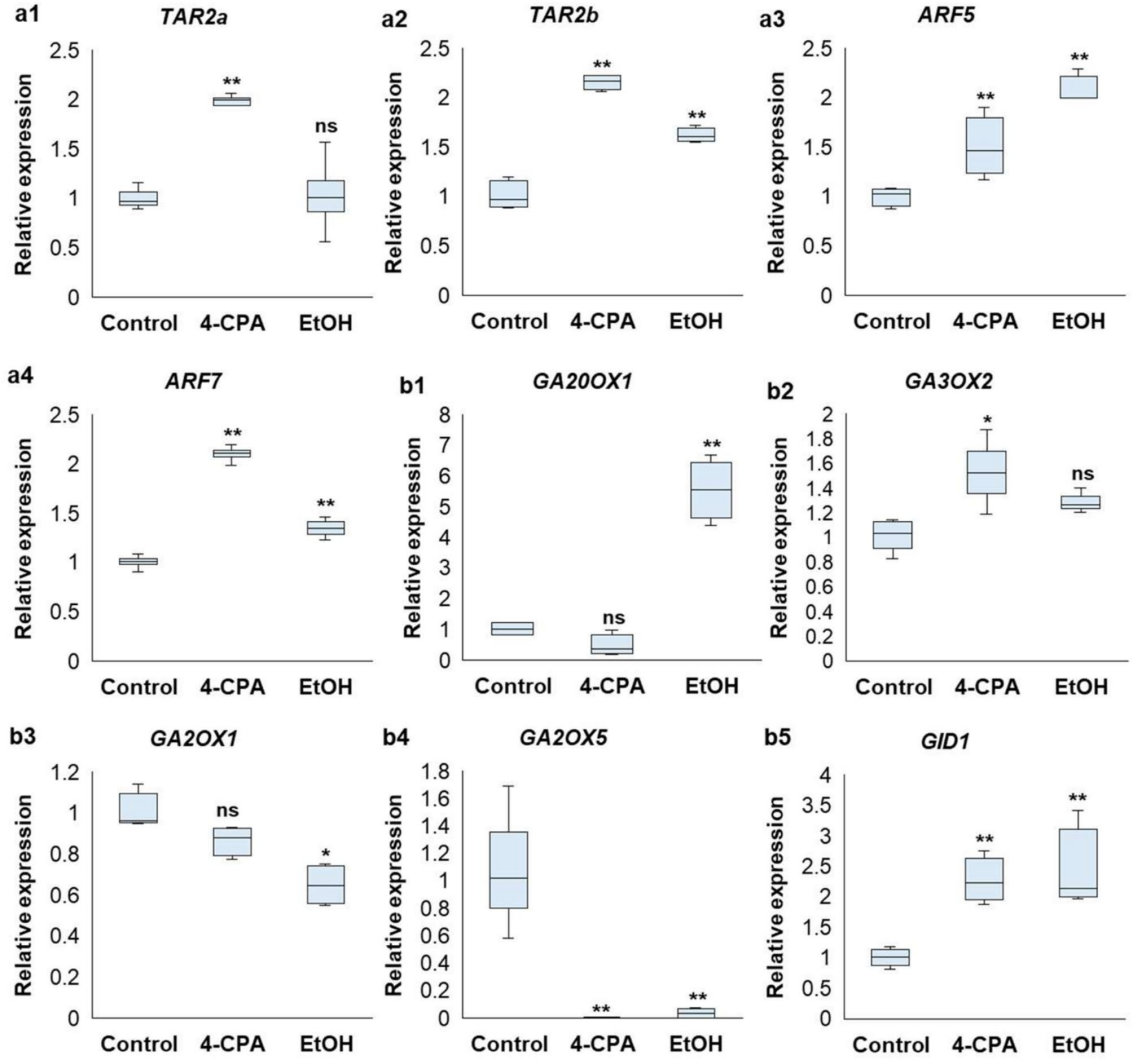
Expression level of auxin signaling-related (**a**) or gibberellin signaling-related genes (**b**) under heat stress treated with 4-CPA or EtOH. The expression level of auxin signaling-related genes, such as *TAR2a*, *TAR2b, ARF5,* and *ARF7*, were measured. The expression level of gibberellin signaling-related genes, such as *GA20OX1, GA3OX2, GA2OX1, GA2OX5,* and *GID1*, were also investigated. Statistical analysis was performed to determine significant differences compared to the control at *p* < 0.05 (*) and at *p* < 0.01 (**) (n ≥ 3).

The tryptophan aminotransferase A (TAA) family produces tryptophan-derived indole-3-pyruvic acid (IPA), while the YUC family functions in converting IPA to indole-3-acetic acid (IAA) in Arabidopsis through a quantification process of IPA. Two gene families encoding key enzymes in the TAA/YUC biosynthesis pathway have been identified in tomatoes, providing new insights into the regulatory mechanisms of auxins during tomato development^25^. The expression level of *TAR2a*, which is categorized in this family, increased significantly following 4-CPA application, and *TAR2b* exhibited significant upregulation under both 4-CPA and ethanol treatments compared to the control plants (Fig. 7a1, a2). Auxin response factor 5 (*ARF5*) is highly expressed in the leaves, flowers, and early immature green fruit in tomatoes, suggesting its role in the development of these tissues and organs. Overexpression of *SlARF5* increases plant height^26^. *SlARF7* exhibits a bidirectional regulatory effect on tomato fruit development by modulating fruit growth-related genes (e.g., *EXPANSIN5*) and mediating crosstalk between auxins and gibberellins (GAs)^27^. The expression levels of both *SlARF5* and *SlARF7* increased significantly with 4-CPA and ethanol application compared to those in the control plants (Fig. 7a3, a4). The increased expression of *TAR2a* and *TAR2b* in tomatoes may enhance auxin biosynthesis, which is associated with growth regulation, developmental changes, and stress responses. Similarly, the upregulation of *ARF5* and *ARF7* suggested improved auxin signaling (Fig. 7a1–a4), significantly affecting fruit development, fruit set, and the potential production of parthenocarpic fruits.

GAs are plant hormones that regulate various aspects of plant growth, including flowering, fruit setting, hypocotyl and stem elongation, root expansion, seed germination, and fruit development^28^. Bioactive GAs are synthesized in the cytosol from GA_12_ and GA_53_ through the activity of two 2-oxoglutarate-dependent dioxygenases (2-ODDs) families, GA 20-oxidases (*GA20ox*), and GA 3-oxidases (*GA3ox*) (Shohat et al., 2021). In this study, the expression levels of two genes related to GA biosynthesis, *GA20OX1* and *GA3OX2*, were analyzed. The expression of *GA20OX1* increased significantly after ethanol application, whereas *GA3OX2* expression increased significantly after 4-CPA treatment. These findings suggested that gibberellin activation occurs in both applications (Fig. 7b1, b2). GA deactivation, primarily catalyzed by GA 2-oxidases (*GA2ox*), reduces the levels of bioactive GAs^29^. The expression levels of the two *GA2ox* genes, *GA2OX1* and *GA2OX5*, were evaluated. The expression of *GA2OX1* decreased following ethanol application, while *GA2OX5* expression decreased under both 4-CPA and ethanol treatments. GA deactivation may be reduced in both applications (Fig. 7b3, b4). GIBBERELLIN INSENSITIVE DWARF1 (*GID1*) is a receptor and nucleocytoplasmic protein that plays a critical role in GA signaling. In the presence of GA, GID1 undergoes a conformational change that enhances its ability to bind to DELLA proteins. DELLA proteins typically suppress GA signaling by inhibiting transcription factors involved in GA-regulated genetic programs^30^. In the present study, the expression level of *GID1* increased significantly with both 4-CPA and ethanol treatments (Fig. 7b5). These results suggested that 4-CPA and ethanol treatment stimulate GA activity by enhancing biosynthesis, reducing deactivation, and promoting GA signaling.

The physiology and abundance of stomata are critical for plant adaptation and acclimatization to diverse habitats under specific environmental conditions. The ability of plants to adjust the number, size, and distribution of stomata on their leaves contributes to their fitness under changing environments. *MUTE* and *FAMA* genes, which encode bHLH-type transcription factors, regulate the initiation and progression of stomatal lineages. These genes promote cell division and fate transitions during stomatal development^31^. In the present study, 4-CPA significantly increased the expression of *MUTE* and *FAMA* under HS conditions (Supplementary Fig. S6). *STOMATAL DENSITY AND DISTRIBUTION 1* (*AtSDD1*) acts as a negative regulator of stomatal density in Arabidopsis, optimizing transpiration and water-use efficiency. In the present study, the expression of *SDD1* decreased significantly following ethanol application, suggesting enhanced stomatal development under stressful conditions (Supplementary Fig. S6). Overexpression of *SDD1-like* gene reduces stomatal density and prevents dehydration in both Arabidopsis and cultivated tomatoes^31^. However, the expression level of *SDD1-like* did not differ between the plants treated with 4-CPA and ethanol and the control plants (Supplementary Fig. S6). Applying 4-CPA and ethanol may enhance stomatal development and function under stressful conditions, potentially contributing to improved stress tolerance in plants.

Fruit quality is determined by several characteristics, including visual characteristics such as fruit size and color, taste characteristics such as sweetness^32^, and nutritional value characteristics such as vitamin C content, which has an antioxidant effect^33^.

**Fig. 8:**
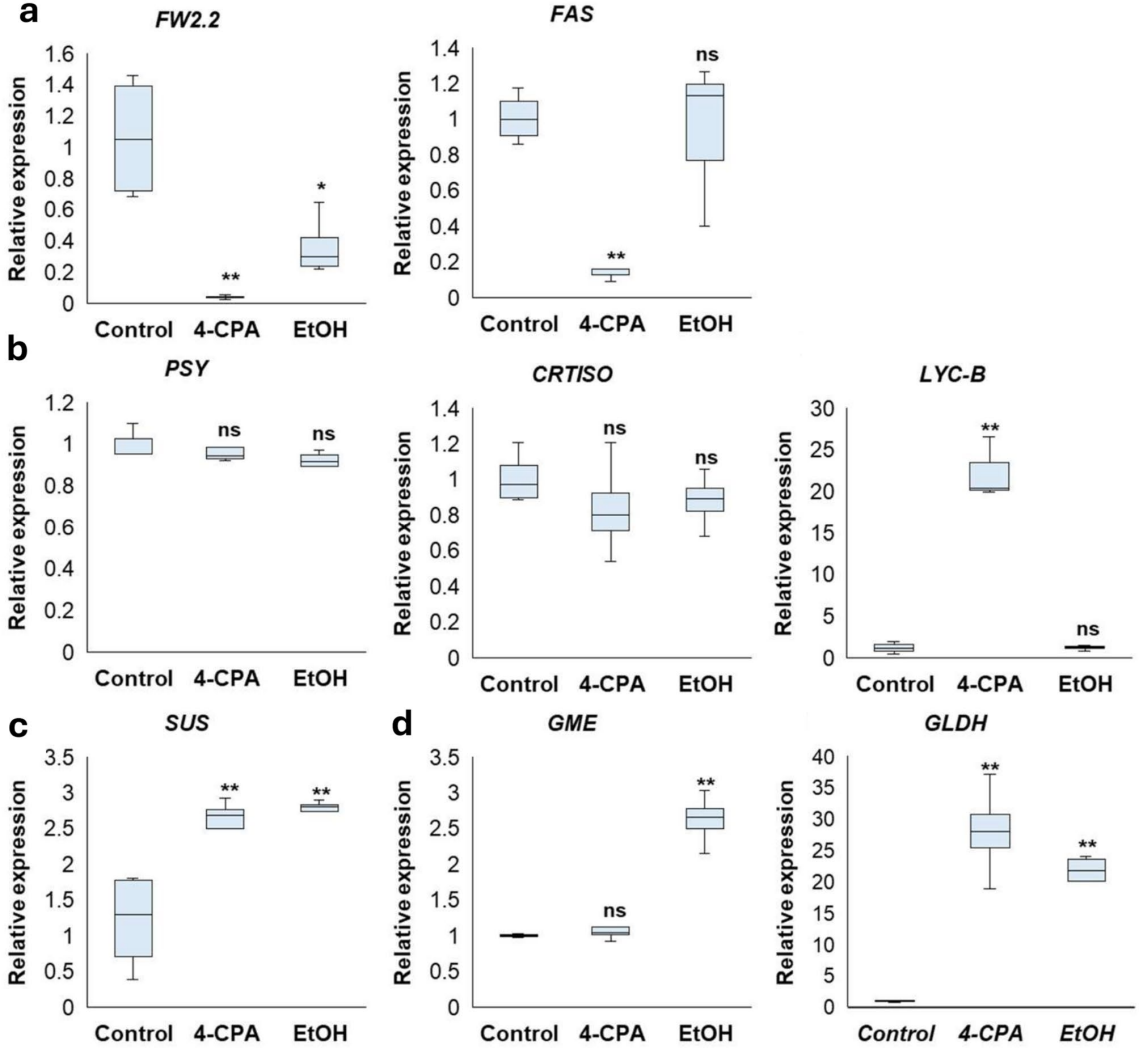
Expression levels of genes involved in fruit size and weight (**a**), fruit color (**b**), sweetness (**c**), and vitamin C synthesis (**d**) were investigated. Statistical analysis was performed to determine significant differences compared to the control at *p* < 0.05 (*) and at *p* < 0.01 (**) (n ≥ 3).

FRUIT WEIGHT 2.2 (FW2.2) is a member of the CELL NUMBER REGULATOR gene family that negatively regulates cell division and affects tomato fruit size and weight in tomato^34^. The fascinated (*FAS*) gene is essential for controlling the number of fruit locules, directly affecting fruit size and shape^35^. The expression of FW2.2 decreased significantly after both chemical treatments compared with that in non-treated *phyA* plants. Only the expression of the *FAS* gene was significantly downregulated in 4-CPA-treated plants compared to that in ethanol-treated and non-treated plants (Fig. 8a), indicating fruit size stimulation after 4-CPA application.

**Fig. 9:**
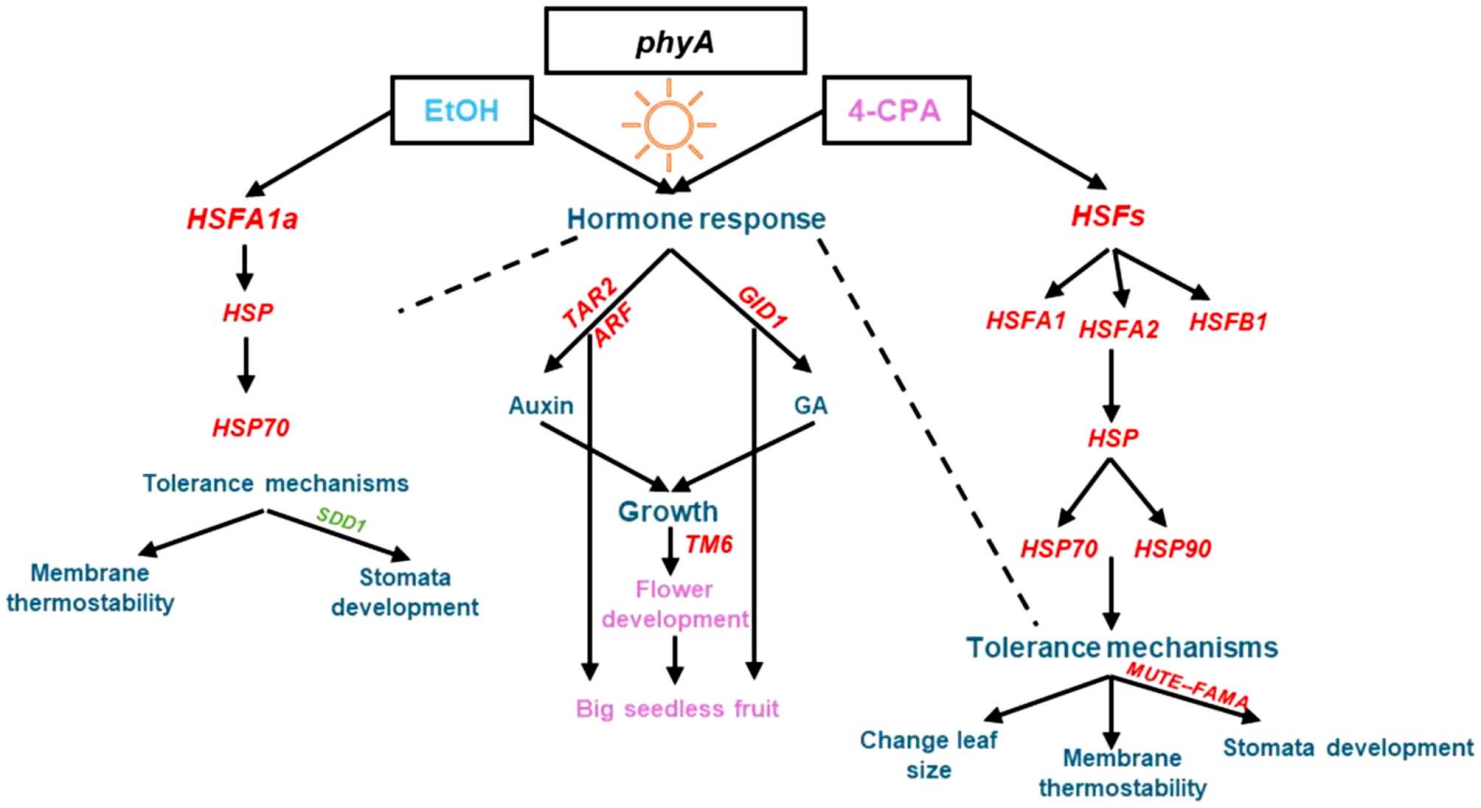
The model of the 4-CPA and EtOH function to improve the *phyA* mutant tolerance to heat stress during the reproductive stage.

Regarding fruit color, the phytoene synthase (*PSY1*) gene regulates the biosynthesis of carotenoids during tomato fruit development and ripening^36^. The carotenoid isomerase (*CRTISO*) gene is responsible for the isomerization of prolycopene to all-trans lycopene in the carotenoid synthesis pathway^37^. The expression levels of both *PSY1* and *CRTISO* genes did not differ markedly between the treated and non-treated *phyA* plants (Fig. 8b). On the other hand, lycopene β-cyclase (LYCB) is a crucial enzyme for producing β-carotene^38^. The 4-CPA-treated plants showed a significantly higher expression level of *LYCB* compared to ethanol-treated and non-treated *phyA* plants (Fig. 8b), indicating the reason for the high β-carotene in tomato fruit treated with 4-CPA (Fig. 2f).

For sweetness, the sucrose synthase (*SUS*) gene is crucial for plant growth and sucrose metabolism^39^. In this study, the expression of the *SUS* gene increased significantly with both 4-CPA and ethanol application compared to that in untreated plants (Fig. 8c), indicating higher sugar accumulation in their fruits.

Regarding the vitamin C content, GDP-Mannose 3′,5′-epimerase (GME) in the D-mannose/L-galactose pathway is a crucial enzyme in the biosynthesis of vitamin C^40^. In the last stage of this pathway, L-galactono-1, 4-lactone is transformed into ascorbate by L-galactono-1,4-lactone dehydrogenase (GLDH)^41^. In this study, the expression level of *GME* increased significantly in ethanol-treated *phyA* plants compared to that in 4-CPA-treated and untreated plants. *GLDH* expression was significantly higher in both 4-CPA- and ethanol-treated plants than in the non-treated *phyA* plants (Fig. 8d), indicating the induction of ascorbic acid after both treatments.

## Discussion

*PHYA* is a key gene involved in sensing far-red light signals and plays a critical role in various developmental stages of plant growth, including flowering and seed development^42^. Additionally, the *PHYA* gene has been linked to stress responses, particularly HS, for which the *phyA* mutant exhibits a tolerance mechanism compared to the Moneymaker WT plants. This tolerance was more pronounced during the vegetative growth stage. However, during the transition to the reproductive stage, tolerance was observed at flowering, but this diminished during the fruiting stage^6^. Application of 4-CPA and ethanol-induced *phyA* tolerance during the flowering and fruiting stages (Fig. 1a). These induced tolerance responses begin by protecting the cell membrane from heat damage. Plants treated with these chemicals exhibited lower MDA and EL accumulation (Fig. 3a, b), which are key indicators of membrane lipid peroxidation and membrane permeability^43^. Furthermore, proline accumulation induction supported *phyA* tolerance activation following the chemical treatment (Fig. 3c). Proline is an important osmoprotectant that stabilizes cellular structures and mitigates heat damage^44^.

The *phyA* plants treated with 4-CPA and ethanol exhibited an increase in stomata number and stomatal pore area compared with non-treated *phyA* plants (Fig. 4c, d). This response suggested an enhanced level of water transpiration, which may facilitate transpiration cooling, a mechanism that helps to mitigate the effects of high temperatures^45^. An increased stomata response in 4-CPA-treated plants was linked to the upregulation of *MUTE* and *FAMA* genes (Supplementary Fig. S6), which are essential for stomata development^46^. Overexpression of either *MUTE* or *FAMA* increases stomatal density and index in tomatoes, with the *MUTE* gene having a particularly significant effect^46^. Conversely, the increase in stomata number and pore areas may also be attributed to the downregulation of the *SDD1* gene (Supplementary Fig. S6). This gene negatively regulates stomatal density and plays a role in optimizing transpiration and water-use efficiently^31^.

The *phyA*-treated plants treated with 4-CPA showed an increased expression of *HSFs* and *HSPs* under HS conditions (Fig. 5). Generally, HS causes growth cessation. Arabidopsis *HSFA1* is considered a master regulator of heat tolerance and its function in tomato plants is similar to that of *HSA1a*^47^. When *HSFs*, particularly *HSFA1*, *HSFA2*, and *HSFB1* are stimulated, they induce the expression of *HSP*, including *HSP70* and *HSP90*. These chaperones are critical in preventing protein denaturation and reducing ROS accumulation^48^. This mechanism was evident in the 4-CPA-treated plants, in which the expressions of *HSFA1a*, *HSFA1b*, *HSFA2*, and *HSFB1* were upregulated. Consequently, the expressions of *HSP*70 and *HSP90* were induced (Fig. 5), leading to reduced lipid membrane peroxidation as indicated by lower MDA accumulation (Fig. 3b). Similarly, ethanol-treated *phyA* plants exhibited higher expression of the tomato master *HSFA1a* than untreated plants, along with a significant upregulation of *HSP70* (Fig. 5). This likely contributed to enhanced cellular protection against membrane lipid peroxidation (Fig. 3b).

Plant growth was also stimulated during the upregulation of *HSFs* and *HSPs* and the protection of cell membranes in the chemically treated plants (Fig. 5). The chemically treated plants exhibited healthier phenotypes, with significant increases in plant height and stem thickness compared to the non-treated *phyA* plants during the reproductive stage (Fig. 1a–c). Generally, plant defense responses to HS are mediated by endogenous phytohormones that stimulate defense mechanisms. Auxins play an important role in HS protection by promoting stem elongation and leaf hyponasty^49^. In the present study, the expression of auxin signaling genes, including *TAR2* and *ARF*, was significantly upregulated in 4-CPA- and ethanol-treated plants (Fig. 7a), indicating increased auxin production. Moreover, GA is involved in plant height, leaf expansion, dry matter accumulation, tissue differentiation, and cell division, and plays a role in tolerance to HS in tomatoes^50^. In this study, the expression of GA biosynthesis genes (*GA20OX1* and *GA3OX2*) was upregulated, whereas that of GA inhibitor genes (*GA2OXs*) was downregulated in 4-CPA- and ethanol-treated plants compared to that in untreated plants. *GA20OX1* expression was markedly increased by ethanol application, and *GA3OX2* expression was significantly upregulated by 4-CPA treatment. Similarly, *GA2OX1* and *GA2OX5* were inhibited by ethanol, with a similar inhibitory effect on *GA2OX5* observed in the 4-CPA-treated plants (Fig. 7b1–4). Additionally, *GID1*, which encodes the gibberellin receptor, was significantly upregulated in response to both chemical treatments (Fig. 7b5). These results suggested that both the auxin and GA pathways were activated following chemical application, enhancing plant growth. The induction of auxin by 4-CPA application was consistent with previous findings that growth regulators such as 4-CPA increase internal IAA levels during the early stages of tomato fruit development^51^. Moreover, the stimulation of *HSP90* in 4-CPA-treated plants (Fig. 5b2) supported the activation of *ARFs* and repression of *Aux/IAA*s transcriptional repressors^52^. The mechanism underlying auxin induction by ethanol treatment remains unclear. However, the induction of *HSP70* may elicit a response similar to *HSP90*, facilitating auxin stimulation. GA induction by both 4-CPA and ethanol treatments appears to result from the crosstalk between the auxin and GA biosynthesis pathways. Specifically, *ARF* genes can stimulate *GA3OXs* expression while repressing *GA2OXs*, thereby enhancing GA production^53^. This interplay was clearly observed in the chemically treated plants (Fig. 7b).

For reproductive organs, it has been reported that the setting of tomato fruit is controlled by successful pollination and fertilization, which trigger fruit development through the auxin and GA signaling pathways^54^. Tomato parthenocarpy can be promoted by GA3 or 2,4-D application, resulting in fruits similar to those formed after pollination^55^. In this study, increased auxin- and GA-related gene expression (Fig. 7) may have enhanced flower and fruit development in 4-CPA-treated *phyA* plants compared to untreated plants. The flower number, fruit set, and fruit weight were significantly improved, and parthenocarpic fruits were observed (Figs. 1d, e, 2b, and Supplementary Fig. S1c). In addition to the roles of auxins and GA in promoting reproductive organ development, the *FA* gene was upregulated in 4-CPA-treated plants, contributing to flowering induction. This was accompanied by the upregulation of the *TM6* gene (Supplementary Fig. S5), regulating normal flower development^56^. Conversely, the ethanol treatment significantly increased *TM6* expression (Supplementary Fig. S5) and partially improved fruit development (Fig. 1e). However, these improvements were not statistically significant compared to those in the non-treated plants.

Although fruit size differed among the treated plants, fruit quality was consistent between the 4-CPA- and ethanol-treated plants when compared to the non-treated plants. Fruit quality was improved by both treatments, as evidenced by increased Brix levels and ascorbic acid content (Fig. 2). The increase in Brix levels can be attributed to GA stimulation following chemical treatment, consistent with previous findings that GA production can enhance sugar content in seedless tomatoes^57^. In addition to the upregulation of the *SUS* gene in *phyA* mutant after being treated with 4-CPA or ethanol (Fig. 8c), this gene catalyzes the reversible reaction of sucrose + UDP ⇄ UDP-glucose + fructose^58^. The increase in the ascorbic acid content was due to the induction of *GLDH* gene expression (Fig. 8d2), which is involved in the transformation of l-galactono-1,4-lactone into ascorbate^41^. This stimulation might be linked to hormone induction (Fig. 7) or the induction of parthenocarpy because parthenocarpic tomato fruits have a higher vitamin C content than seeded fruits^59^.

Along with Brix values and vitamin C content, fruit color is a critical factor in determining fruit quality. Lycopene and *β*-carotene are the key pigments responsible for tomato fruit color^60^. Fruits from 4-CPA-treated plants exhibited high *β*-carotene and low lycopene content (Fig. 2e, f), resulting in an orange-colored tomato fruit (Fig. 2a). This effect may be attributed to 4-CPA’s role as a synthetic auxin, as previous studies have shown that exogenous auxin application in tomatoes can increase *β*-carotene content and decrease lycopene levels at the later stages of development compared to non-treated plants^60^. Induction of *LYC-B* gene expression after 4-CPA application (Fig. 8b3) is involved in the *β*-carotene stimulation because of its function as a crucial enzyme for β-carotene production^38^.

Ethanol can both increase and decrease reactive oxygen species (ROS) in plants, depending on the context. Ethanol can initially increase ROS production^61^, but under stress conditions, it can also decrease ROS levels^14,17^. Increase in ROS enhances stomatal closure^62^. Stomatal aperture under heat stress conditions was observed in this study, ROS level may decrease, and stomata were opened as shown in previous report, in which ethanol suppressed ROS accumulation in high light condition^17^. Furthermore, auxin generally works as a positive regulator for stomatal opening^63^. These effects probably promote stomatal opening in this study.

In summary, this study reveals a strategy for improving heat stress tolerance in tomato by utilizing exogenous application of 4-CPA and ethanol to activate key hormonal and stress-responsive genes in the *phyA* mutant tomato plants. These findings provide an insight into the molecular and physiological mechanisms underlying heat tolerance and offer tools for developing resilient tomato cultivars through chemical treatment-based approaches. Furthermore, this study demonstrated that applying 4-CPA to flower clusters induces various heat tolerance mechanisms, enhancing the development and fruit formation in *phyA* mutant plants. Additionally, ethanol application enhanced plant tolerance to HS, as evidenced by physiological responses, while improving fruit quality (Fig. 9).

## Materials and methods

### Plant materials and growth conditions

The *phyA* mutant seeds (*Solanum lycopersicum* cv. Moneymaker) were grown in soil or Rockwool cubes and incubated at 25 °C under a long-day photoperiod (16 h light/8 h dark) until the flowering stage. Plants were exposed to heat stress (HS) under two different conditions after flowering: a constant high temperature of 37 °C and fluctuating high temperatures under greenhouse conditions (from July to October). The temperatures were approximately 50 °C and 30 °C in sunny midday and night, respectively, from July to mid September.

### Chemical treatment

Three different groups were designed for the chemical treatment. The first group consisted of *phyA* mutant without any treatment, the second group was sprayed with 4-CPA at 20 ppm, and the third group was sprayed with 20 mM ethanol. These concentrations of 4-CPA and ethanol were based on the result of tomatoes; application of 4-CPA at 20 ppm increased numbers of fruits and plant yield^8^ and application of 20 mM ethanol enhanced heat tolerance of tomato^11^. The chemicals were dissolved into water. For the 4-CPA application, flower clusters were targeted and sprayed once per week. From 10 to 20 mL of 4-CPA was sprayed for one flower cluster. In the third group, ethanol was sprayed onto the whole plant once per week for one month until the first fruit was set. From 50 to 100 mL of ethanol was sprayed for each whole plant. All the groups were subjected to HS conditions until the plants reached the flowering stage.

### Morphological phenotype

The morphological characteristics of the tomato plants’ vegetative organs, including plant stem height and stem thickness, were measured. Plant height was measured using a ruler, and stem thickness was determined using a digital caliper. The number of flowers and clusters and the percentage of developed flowers and clusters under HS conditions were recorded as the floral characteristics. The average fruit fresh weight (FW), length, diameter, fruit set percentage, and parthenocarpy occurrence were recorded as the fruiting characteristics to evaluate the treatment responses under HS conditions.

### EL measurement

EL in tomato leaves was analyzed as previously described^6^. Briefly, the leaf surface was washed with Milli-Q water (MQ) to remove ions from the surface and the leaves were submerged in a tube filled with MQ to cover all parts of the leaf. The tube was incubated in a water bath at 43 ± 1 °C for 1 h. The initial ionic conductivity (IC1) was measured after cooling to room temperature (23 ± 2 °C). The samples were then autoclaved at 121 °C for 10 min, cooled to room temperature, and the second ionic conductivity (IC2) was measured. A conductivity meter (Lutron Electronics Co., Inc., Coopersburg, PA, USA) was used to measure the ionic conductivity. The percentage of EL was calculated using the following formula: EL (%) = IC1 / IC2 × 100.

### Measuring proline and MDA levels

Proline content in the leaf samples was determined as previously described^64^. The absorbance at 520 nm (A_520_) was measured using a DU-800 spectrophotometer (Beckman Coulter, Inc., Brea, CA, USA). Proline concentration was calculated using the following equation:

$${\text{Proline }}\left( {{{\mu {\rm mol}}}/{\text{g}}} \right) = \frac{{{\text{A }}520{ }\left( {{{\mu {\rm g proline}}}/{\text{mL}}} \right) \times {\text{Toluene amount}}\left( {{\text{mL}}} \right)}}{115.13}/\frac{{{\text{Sample FW}}\left( {\text{g}} \right)}}{5}$$

The MDA levels were measured as described^43^ and calculated using the following formula:

$${\text{MDA }}\left( {{{\mu {\rm mol}}}/{\text{L}}} \right) = \left[ {{6}.{45 } \times \, \left( {{\text{A}}_{{{532}}} {-}{\text{A}}_{{{6}00}} } \right) \, - \, 0.{56 } \times {\text{ A}}_{{{45}0}} } \right]$$

### Microscopic analysis of stomata

Fresh leaflets were collected to prepare the leaf samples for stomatal analysis. A piece of thick tape was applied to the upper surface of the leaflet and gently removed to peel off the epidermis, thereby exposing a thin transparent layer of surface cells. The epidermal layer was then placed on a microscope slide, and the leaflet was carefully trimmed using a sharp scalpel. One drop of water was added to the sample, and a coverslip was placed over it. The stomatal number, pore length, and aperture were examined using an Olympus BX50 microscope (Olympus, Tokyo, Japan). As described previously^65^, the stomatal number was determined under 40 × magnification with a counting area of 92.7 mm^2^, whereas the pore length and aperture were measured under 100 × magnification. ImageJ software was used for measuring.

### RNA isolation and quantitative reverse transcription-polymerase chain reaction (qRT-PCR)

Total RNA was extracted from leaves after 4 weeks of exposure to HS to investigate heat- and stress-responsive genes during the flowering stage. Total RNA was isolated from the three groups of plant samples using TRIzol reagent (Thermo Fisher Scientific, Waltham, MA, USA) following the manufacturer’s instructions. A total of 2 µg of RNA was used to synthesize cDNA using a high-capacity cDNA reverse transcription kit (Thermo Fisher Scientific). The primers used for the real-time PCR are listed in Table S1. RT-PCR amplification and detection were performed using THUNDERBIRD SYBR qPCR Mix (Toyobo, Osaka, Japan) on a 7900HT real-time PCR system (Applied Biosystems/Thermo Fisher Scientific). The relative transcript abundance was calculated using the comparative C_T_ method. The ΔC_T_ of WT under HS as a subthreshold factor in the ΔΔC_T_ subtraction formula for comparison with the *phy* treatment was calculated as follows: ΔΔC_T_ (ΔC_T_ − ΔC_T, WT (stress)_). The tomato *EXPRESSED* gene was used as an endogenous control for the gene expression analysis^66^.

### Pollen fertility and pollen tube growth test

Newly blooming flowers were collected daily from plants grown under HS conditions to extract pollen grains. Anther cones were isolated from the flowers and left to dry for 3–4 h. Each anther cone was divided into 2–3 parts, and pollen grains were extracted by gently tapping the cones. Pollen was collected in a tube for subsequent microscopic analysis of pollen fertility and pollen tube growth.

Pollen fertility was analyzed by staining pollen grains with potassium iodide, as described previously^67^. The stained pollen was observed under an Olympus BX50 microscope. The fertile pollen grains were manually counted across at least four microscopic fields.

The flowers were emasculated 1 day before flower opening. Manual cross-pollination using pollen extracted after the stress treatment was performed the following day. After 24 h, the pistils were collected and immersed in a fixing solution (3:1 ethanol:acetic acid) for 12 h, followed by immersion in 75% ethanol for 6–8 h, and transfer to a softening solution (5 M NaOH) for 12–16 h. An aniline blue working solution was prepared 1 day in advance by diluting 0.01% (v/v) aniline blue stock solution with 0.1 M K_2_HPO_4_ (pH 10) at a 1:10 ratio and storing it at 4 °C in the dark overnight. The pistils were then transferred to the aniline blue working solution for 24 h, mounted on a glass slide with glycerol as the mounting agent, and flattened by firmly pressing the cover glass. The sections were observed under an Olympus BX50 UV microscope.

### Lycopene and *β*-carotene contents

Fresh tomatoes (1 g) were mixed with 1 mL of acetone: hexane (4:6) and placed on ice for 10 min to measure lycopene content. The mixture was centrifuged at 1,370 × *g* for 10 min and the absorbance was measured at 663, 645, 505, and 453 nm^68^ using a DU-800 spectrophotometer (Beckman Coulter). The following formula was used:

$$Lycopene \left( {mg/mL} \right) = - 0.0458 A663 + 0.204 A645 + 0.372 A505 - 0.0806 A453$$

Fresh tomatoes (1 g) were mixed with 1 mL of acetone and placed on ice with shaking for 15 min. To measure *β*-carotene content. The mixture was vigorously mixed for 10 min, centrifuged at 1370 × *g* for 10 min, and the absorbance was measured at 449 nm using a DU-800 spectrophotometer (Beckman Coulter, Inc). Using the following formula, Y = aX + b, where Y is the response (Absorbance), X is the concentration of *β*-carotene, a is the slope, and b is the intercept of the standard curve^69^.

### Ascorbic acid content

Ascorbic acid content was measured as described previously^70^. Tomato fruits (1 g) were homogenized and mixed with 2 mL of 5% metaphosphoric acid (w/v). After centrifugation at 12,000 × *g* for 3 min, the supernatant was collected as the crude extract. Total ascorbic acid content was measured using a reflectometer (RQflex Plus 10).

### Statistical analyses

Analysis of variance was performed using the ASTATA database to analyze the quantitative data, with means compared using Duncan’s multiple range test (*p* < 0.05) or Tukey HSD post-hoc test.

## Supplementary Information

Supplementary Information.

## Data Availability

Data available under request to the corresponding author.
