# Supplementary material for: Application of 4-CPA or ethanol enhances plant growth and fruit quality of phyA mutant under heat stress
Source: Sci Rep. 2025 Sep 12;15:32388. doi: 10.1038/s41598-025-17929-8 (PMC12432177; doi:10.1038/s41598-025-17929-8)
Supplement: Supplementary file 1 — Supplementary Information. [file 41598_2025_17929_MOESM1_ESM.pdf]

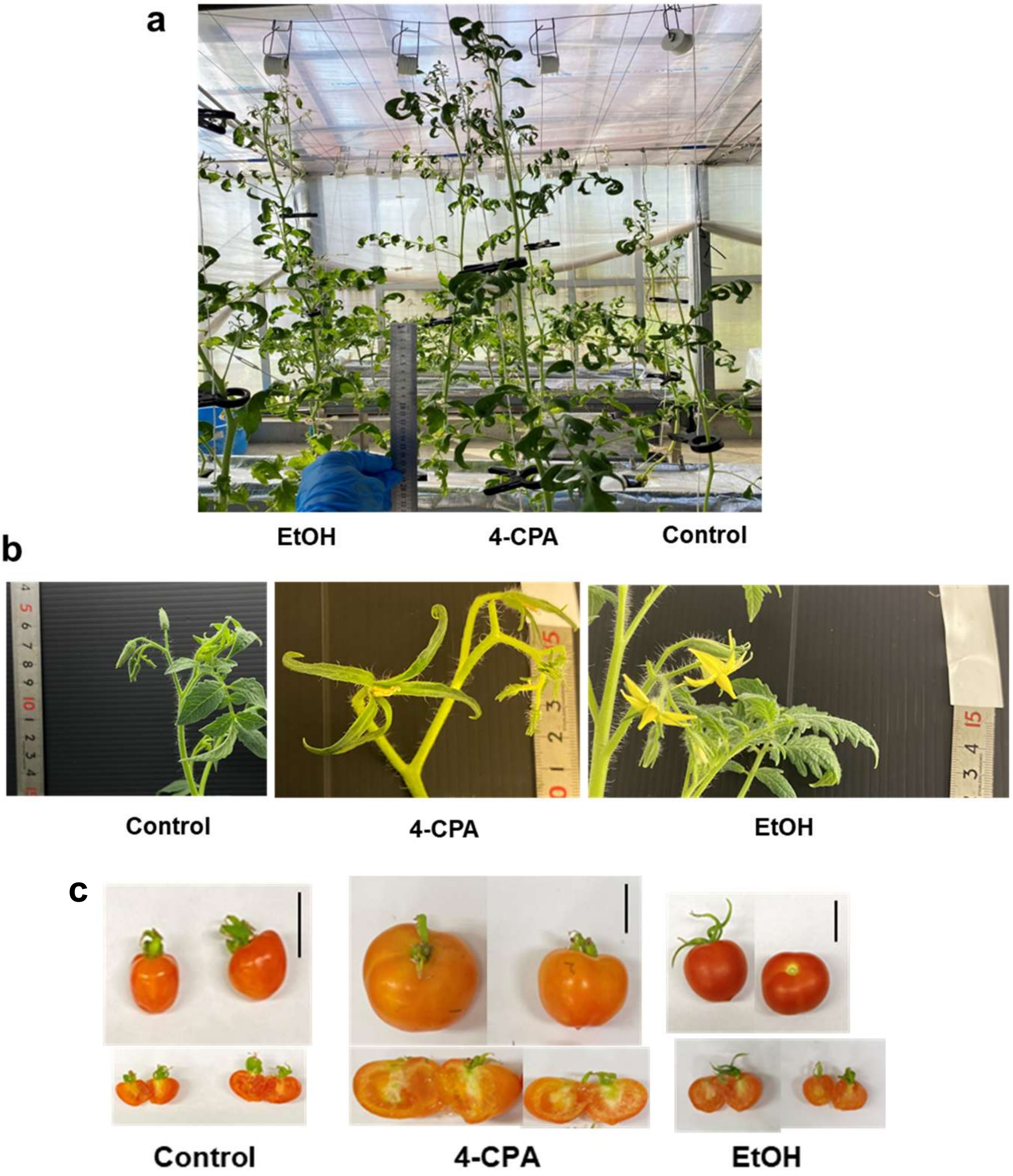

Fig. S1. Morphological response under greenhouse conditions treated with 4-CPA or ethanol (EtOH). (a) Plant phenotype under greenhouse conditions. (b) Flowers of plants grown in greenhouse treated with 4-CPA or EtOH. (c) Tomato fruits, which showing parthenocarpy.

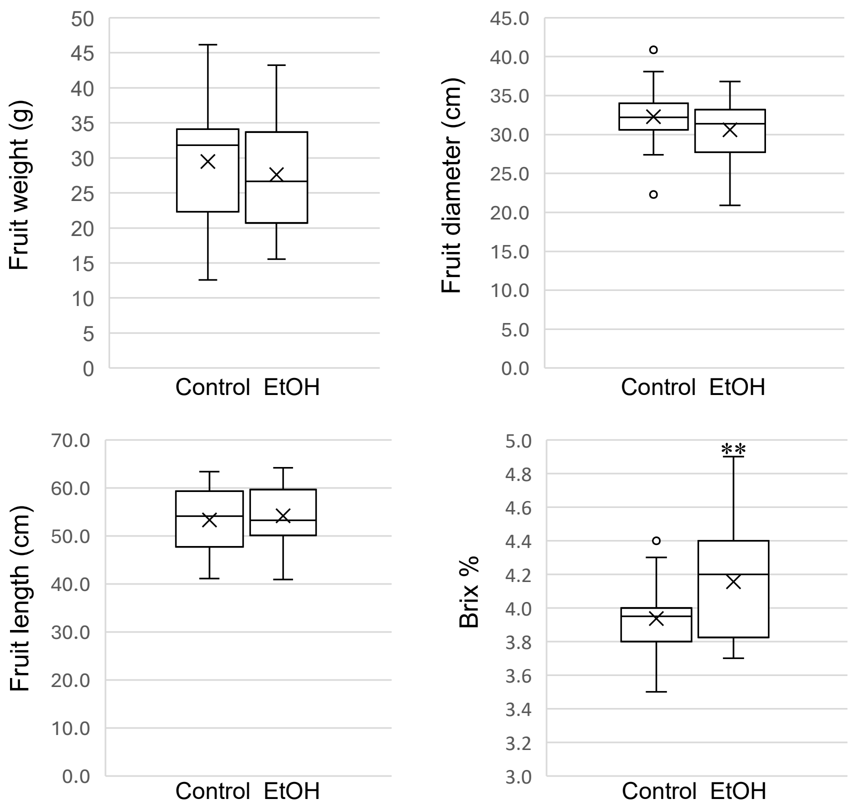

Fig. S2. Fruit quality of another tomato cultivar Sicilian Rouge treated with EtOH. Morphological parameters, such as fruit weight, fruit diameter, and fruit length were investigated. Brix was measured by a refractometer. Statistical analysis was performed to determine significant differences compared to the control at $p < 0.01$ (**) by Student's t-test ($n \geq 30$).

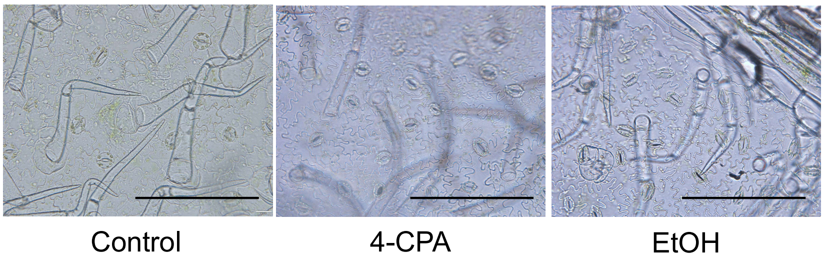

Fig. S3. The image are representative microscopic views of 92.7 mm^2 area. Bars indicate 5-mm length.

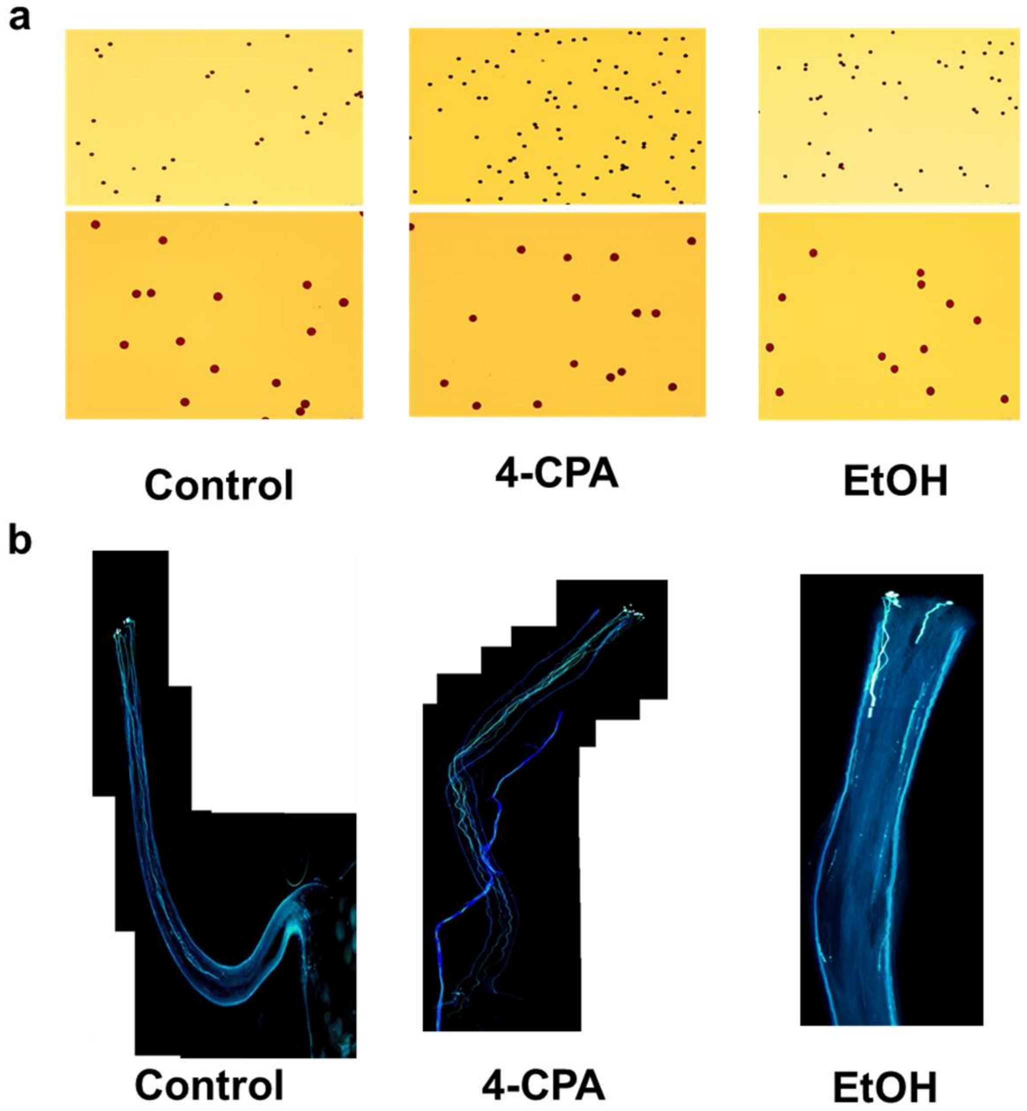

Fig. S4. Microscopic analysis of pollen under heat stress treated with 4-CPA or EtOH. (a) Pollen fertility was analyzed by staining the pollen grain with potassium iodide. The stained pollen was observed under Olympus BX50 microscope. (b) *In vivo* pollen tube growth. One day prior to the flower opening, flowers were emasculated. The following day, manual cross-pollination was performed using pollen extracted after stress treatment. After 24 h, the pistils were collected and immersed in a fixing solution (3:1 ethanol: acetic acid) for 12 h, followed by immersion in 75% ethanol for 6-8 h and transfer to a softening solution consisting of 5M NaOH for 12-16 h. The aniline blue working solution was prepared one day in advance by diluting 0.01% (v/v) aniline blue stock solution with 0.1M K_2HPO_4, pH10 at 1:10 ratio and storing it at 4 ºC in the dark overnight. The pistils were then transferred to the aniline blue working solution for 24 h, mounted on a glass slide with glycerol as a mounting agent, and flattened by firmly pressing the cover glass. The sections were observed under Olympus BX50 UV microscope.

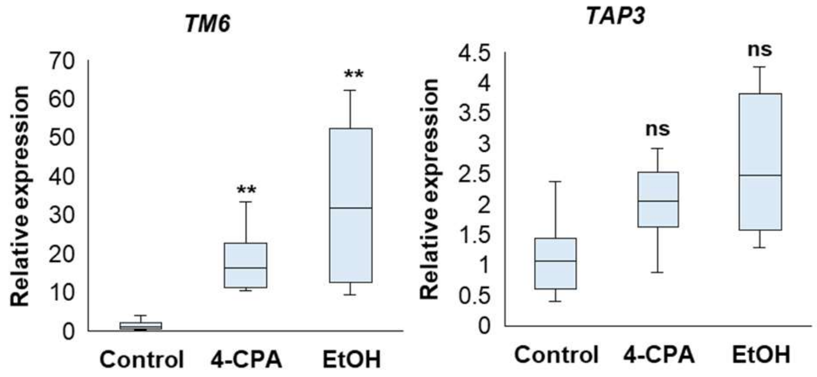

Fig. S5. Expression level of *TM6* and *TAP3* genes for floral development. Statistical analysis was performed to determine significant differences compared to the control at $p < 0.01$ (**) ($n \geq 3$).

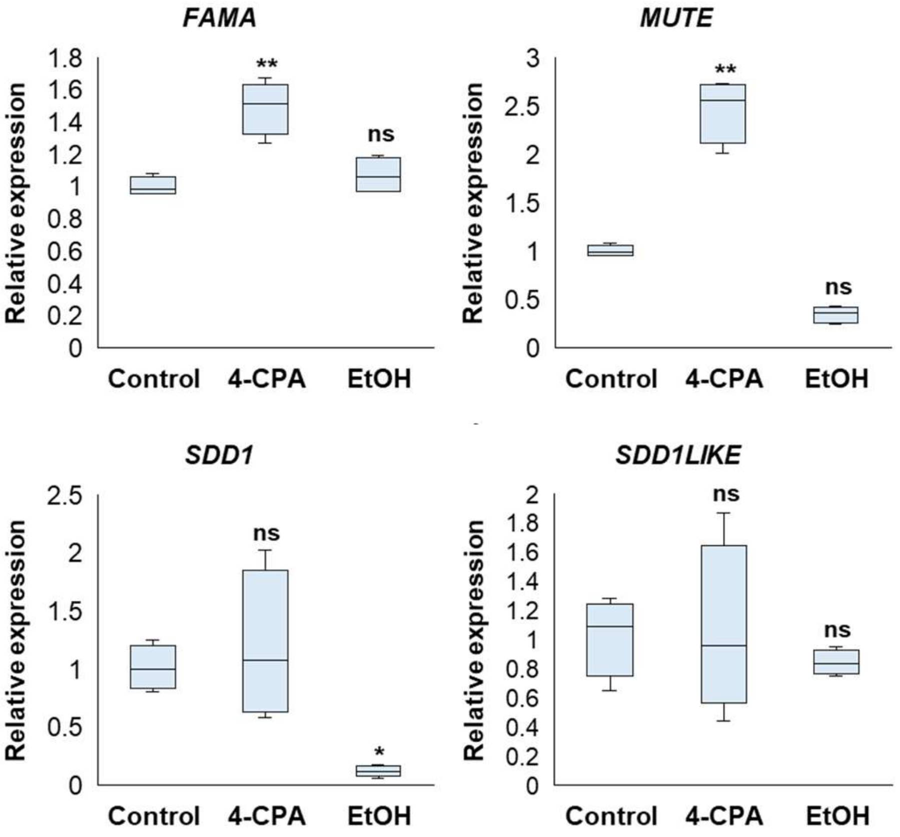

Fig. S6. The expression of stomata development-related genes, including *FAMA*, *MUTE*, *SDD1,* and *SDD1-like* under heat stress treated with 4-CPA or EtOH. Statistical analysis was performed to determine significant differences compared to the control at $p < 0.05$ (*) or at $p < 0.01$ (**) (n≥3).

Table S1. Primers are used for RT-qPCR for gene expression analysis.

Target gene	Sequence (5'-3')	
SlHSFA1a	Forward	GTCGAGGTTGGGAAATTTGG
	Reverse	TGTTGTCTGAGCCTAACCAG
SlHSFA1b	Forward	CAACAACTTCTCCAGCTTCG
	Reverse	TAAGAAATCCCTCGTTCGCA
SlHSFA2	Forward	CCAGTGCTACAGGAAGTGAT
	Reverse	TTCATTGTCCATTGCAGCAG
SlHSFB1	Forward	GTGGTGGACGTGGTAAAATG
	Reverse	TACTCTGCTCCAACCTCAAC
SlHSFA4a	Forward	TCCTTAACCAACGTGTCCAA
	Reverse	TGGCAACCTTCTCTTCCTTT
SlHSFA5	Forward	CACAGTCATAGTCACCCTCC
	Reverse	TGGAGTTTTGCAGCAGATTG
SlHSP70	Forward	TGCCCAAAGATGAGGTTGAT
	Reverse	ATTCTGCTTGATTCTTCGCG
SlHSP90	Forward	AGCGGTATTGGCATGACTAA
	Reverse	TACGCAGAGTAGAAACCCAC
SlARF5	Forward	AGTAACAGCGGGAGCACAGTGATTG
	Reverse	TTACATTGCTTGATGATGCACCACC
SLARF7	Forward	GCAGATGAGCTTGAATGGGG
	Reverse	CCACGCATTACCACTGTCAG
SlTAP3	Forward	TCAGACAGAGGATGGGAGAA
	Reverse	AGCTTCAGAGAATTGTCCACA
SlTM6	Forward	CTCCATTCGCAAATGCCACAGC
	Reverse	GAAGGCGAGGAACTTAGAAGAGCAA
SlExpressed	Forward	GCTAAGAACGCTGGACCTAATG
	Reverse	TGGGTGTGCCTTTCTGAATG
SLFAMA	Forward	ACCAGAGTTCGTTAGGGTTTT
	Reverse	ATTTAGGCCCAAAATCCGCG
SLMUTE	Forward	AGCAGTGGAGAGAAACAGGA
	Reverse	TGCTTGATCACCCCTTTTGA
SlTAR2a	Forward	CCTACGAGCACCCAGAGAAA
	Reverse	GTCTCCATCGCTTTGCCATT

SlTAR2b	Forward	TCAGGGGAGCTGTGTTCAAT
	Reverse	TATGTCATGGTCTGCCCTCG
SlGID1	Forward	TCGCCCCTTTCTCATTGCTA
	Reverse	CTCCCTCTCGACACACTGTT
SlGA20OX1	Forward	CTTGTTGGGGAAGCGTGTC
	Reverse	GCGGCAACTCAAAGAACGTA
SlGA3OX2	Forward	GAGTGTTTTACATCGGGCGG
	Reverse	TTTCGACAGGGGTGAGACTC
SlGA2OX5	Forward	CCTGCTTCCCCTTACGGTTA
	Reverse	GAGTTTGAGGAATGGTGACGG
SlGA2OX1	Forward	TGTTGGTGACTCATTGCAGG
	Reverse	TGACACCCTGGCTTTCAAAC
SlSDD1	Forward	CCAGGTGTCAACATTATCGC
	Reverse	GCAATGCCACTAACATGAGG
SlSDD1like	Forward	TTGGAGGAATGGTAATAGGA
	Reverse	TGAGAATTGAAGGATCAGTATAG
SlAPX1	Forward	CTCTCCTTTGTGATCCTGCT
	Reverse	CAGAAAGCTTCAAGTGAGCC
SlAPX2	Forward	TCATGGTGCAAACAATGGTC
	Reverse	ACTTCAACAGCAACAACACC
SlCAT1	Forward	ATCAGGGACATTCGTGGTTT
	Reverse	TCAGGGAACGACTTAGCATC
SlCAT2	Forward	GAGGTGGATTATTTGCCCTCGAGGTTTG
	Reverse	TACCTCTCCCCTGCCTGTTTGAAGTTG
SlSOD	Forward	GGATTTGTAGCAGCCATTGG
	Reverse	TGAAGCCAAAGTCAGCAATG
SlLYC-B	Forward	TCTATTAGCCGCCTCGTGAG
	Reverse	TACCTTCCTCGACCCTACCA
SlPSY1	Forward	CTGAGATCTACCAATGAGTTAG
	Reverse	TCTCGGGAGTCATTAGCATAG
SlCRTISO	Forward	GAGATCCTGGGTTGCTGTCT
	Reverse	TCCGCCAAAATGTCTGTCAC
SlGME	Forward	ATGTGGGGAGATGGAAAGCA
	Reverse	TTCCGATGTTCACAGGCTCT
SlGLDH	Forward	GTGGAGGAGCTTGAAGGGAT

	Reverse	GCTCGTGTTAACCCAATCCC
SlFW2.2	Forward	GCCCTTGTATCACCTTTGGAC
	Reverse	GCAATCCTGTCAATCCCAGC
SlFAS	Forward	TCCTTCTGCGTACAACCGAT
	Reverse	ATTTGTTGCCCTCCAGCTTG
SlSUS2	Forward	CTTTGGAGGCCGAAATGCTT
	Reverse	AGCATCAGGTATCAGTCGGG
